# Differential Mortality and High Viral Load in Naive Pacific Oyster Families Exposed to OsHV-1 Suggests Tolerance Rather than Resistance to Infection

**DOI:** 10.3390/pathogens9121057

**Published:** 2020-12-17

**Authors:** M. Victoria Agnew, Carolyn S. Friedman, Christopher Langdon, Konstantin Divilov, Blaine Schoolfield, Benjamin Morga, Lionel Degremont, Arun K. Dhar, Peter Kirkland, Brett Dumbauld, Colleen A. Burge

**Affiliations:** 1 Institute of Marine and Environmental Technology, University of Maryland Baltimore County, Baltimore, MD 21202, USA; magnew1@umbc.edu; 2 School of Aquatic and Fishery Sciences, University of Washington, Seattle, WA 98195, USA; carolynf@uw.edu; 3 Department of Fisheries and Wildlife, Coastal Oregon Marine Experiment Station, Hatfield Marine Science Center, Newport, OR 97365, USA; chris.langdon@oregonstate.edu (C.L.); divilovk@oregonstate.edu (K.D.); Blaine.Schoolfield@oregonstate.edu (B.S.); 4 Ifremer, SG2M, LGPMM, 17390 La Tremblade, France; Benjamin.Morga@ifremer.fr (B.M.); lionel.degremont@ifremer.fr (L.D.); 5 Aquaculture Pathology Laboratory, School of Animal and Comparative Biomedical Sciences, University of Arizona, Tucson, AZ 85721, USA; adhar@arizona.edu; 6 NSW Department of Primary Industries, Elizabeth Macarthur Agricultural Institute, Menangle, NSW 2568, Australia; peter.kirkland@dpi.nsw.gov.au; 7 Hatfield Marine Science Center, USDA-ARS, Newport, OR 97365, USA; brett.dumbauld@usda.gov

**Keywords:** OsHV-1, µvars, oyster, virus, tolerance, virulence, qPCR

## Abstract

Pacific oysters, *Crassostrea gigas*, are one of the most productive aquaculture species in the world. However, they are threatened by the spread of Ostreid herpesvirus-1 (OsHV-1) and its microvariants (collectively “µvars”), which cause mass mortalities in all life stages of Pacific oysters globally. Breeding programs have been successful in reducing mortality due to OsHV-1 variants following viral outbreaks; however, an OsHV-1-resistant oyster line does not yet exist in the United States (US), and it is unknown how OsHV-1 µvars will affect US oyster populations compared to the current variant, which is similar to the OsHV-1 reference, found in Tomales Bay, CA. The goals of this study were to investigate the resistance of *C. gigas* juveniles produced by the Molluscan Broodstock Program (MBP) to three variants of OsHV-1: a California reference OsHV-1, an Australian µvar, and a French µvar. This is the first study to directly compare OsHV-1 µvars to a non-µvar. The survival probability of oysters exposed to the French (FRA) or Australian (AUS) µvar was significantly lower (43% and 71%, respectively) than to the reference variant and controls (96%). No oyster family demonstrated resistance to all three OsHV-1 variants, and many surviving oysters contained high copy numbers of viral DNA (mean ~3.53 × 10^8^). These results indicate that the introduction of OsHV-1 µvars could have substantial effects on US Pacific oyster aquaculture if truly resistant lines are not achieved, and highlight the need to consider resistance to infection in addition to survival as traits in breeding programs to reduce the risk of the spread of OsHV-1 variants.

## 1. Introduction

Aquaculture provides a substantial contribution to food production throughout the world. Farming of aquatic species contributed over 80 million tons of food in 2017, resulting in a revenue of ~USD 230 billion globally [1]. In this lucrative market, the health of aquaculture species is vital to the success of the industry; however many of these species are threatened by disease. Disease outbreaks have historically devastated aquaculture industries and caused harsh economic losses, for example: white spot syndrome virus in shrimp [2], infectious salmon anemia virus [3], and a multitude of diseases in mollusks including dermo and MSX disease [4]. Distinct periods of increase in marine disease have been reported for multiple species over the years, including mollusks [5,6], which make up ~21% of aquaculture production [1]. Disease dynamics are driven by host–pathogen interactions and can depend heavily on environmental influence, especially changes in temperature and salinity [7]; consequently, an increased frequency of disease outbreaks is likely to continue under changing environmental conditions in the coming years [8,9]. Understanding and managing the risk of disease for primary economic aquaculture species therefore becomes very important for the future success of the aquaculture industry [10].

Globally, Pacific oysters (*Crassostrea gigas*) are among the top 30 principal aquaculture species with a total revenue of ~USD 1 billion in 2017 [1]. They are also of particular importance in the United States (US), where *C. gigas* sales accumulated USD 89 million and contributed to 31% of total oyster sales in 2018 [11]. However, the spread of the viral pathogen Ostreid herpesvirus 1 (OsHV-1) has threatened this industry since its detection in the 1990s. OsHV-1 was discovered in association with mass mortalities (80–100%) of larval and juvenile *C. gigas* in France [12,13] and larval *C. gigas* (60–100%) in New Zealand hatcheries [14]. In the US, OsHV-1 is responsible for recurring summer mass mortalities of juvenile oysters in Tomales Bay, CA, and likely has been since 1993 [15,16,17]. In 2008, a genetically distinct variant of OsHV-1 was discovered in France [18]. This new variant, referred to as OsHV-1 microvariant (µvar), is characterized by having deletions in a microsatellite region and multiple polymorphisms compared to the reference genome sequenced from the original outbreak in France [18,19,20]. This OsHV-1 µvar caused high mortality and was detected at a higher prevalence in batches of oysters compared to past outbreaks with OsHV-1 [18,21].

Since the discovery of the OsHV-1 µvar in France, genetically similar variants, referred to collectively as “µvars”, have so far been detected in multiple European countries [22], Asia [23,24,25], Australia [26], and New Zealand [27,28]. Historically, only one variant similar to the reference OsHV-1 [15,29] has been detected in the US in Tomales Bay, California (OsHV-1 TB), where it causes annual mortalities of *C. gigas* [15,16,17,22]. However, in November of 2018 an OsHV-1 µvar was detected in San Diego, CA, in association with mass mortalities of *C. gigas* [30]. The emergence of these variants globally suggests that OsHV-1 µvars are spreading through unregulated sources, although animal transfers have been linked to new detections [22]. Although the actual method(s) of pathogen transfer is still unclear, such sources could include contaminated equipment, ballast water, bivalves attached to ship hulls, particle attachment, live animal markets, or other vector species besides *C. gigas* [25,31,32,33,34]. Based on the pattern of rapid emergence historically seen with OsHV-1 µvars, detection of this virus in the US makes the transmission of OsHV-1 µvar to additional areas outside of San Diego almost inevitable, threatening naive oyster farms along the West coast. 

Breeding oyster lines for resistance to pathogens has become a useful tool to mitigate losses [35]. Dermo disease (caused by *Perkinsus marinus*) and MSX disease (caused by *Haplosporidium nelsoni*) have both historically devastated Eastern oyster (*C. virginica*) aquaculture in the US [36]. Selective breeding of Eastern oysters has led to dermo and MSX resistant oyster lines, which are almost exclusively used in US aquaculture today [37,38]. This strategy has also been valuable in mitigating OsHV-1 infection in several countries including France, Australia, and New Zealand where selective breeding has successfully decreased the mortality of oysters exposed to OsHV-1 and its variants following viral outbreaks [39,40,41,42,43]. Studies also indicate that careful selection of oyster lines or stocks can reduce mortalities in oysters affected by OsHV-1 TB in the US [16,17]. However, since the majority of the hatcheries in the US are located outside of the disease endemic zone, true “selective breeding” (i.e., breeding survivors of mortality events) for OsHV-1 resistance is not currently feasible. Additionally, the import of selected lines from selective breeding programs in a disease-endemic location would not be possible due to restrictions on the transportation of potentially infected individuals. Nevertheless, some Pacific oyster stocks and family lines grown in the US industry likely have alleles for OsHV-1 resistance and thus can be bred for resistance. A lack of OsHV-1-resistant lines in the US further highlights the need to begin identifying oyster families with increased survival and/or disease resistance prior to potential OsHV-1 µvar introductions to help reduce catastrophic losses to this industry. 

Observational data indicate that the OsHV-1 µvar may have increased virulence compared to the reference OsHV-1 [18] and that virulence can vary among different OsHV-1 µvars [44,45]. However, a direct comparison of the effects of OsHV-1 µvar and a reference variant has yet to be investigated in a single study, so it is unknown how a potential emergence of an OsHV-1 µvar would affect Pacific oysters if it were to spread within the US. Multiple OsHV-1 variants have emerged since its original detection in the 1990s, and these variants have spread rapidly across borders; therefore, it is crucial that an OsHV-1-resistant oyster line can withstand exposure to multiple OsHV-1 variants, not just a single OsHV-1 µvar. 

The Molluscan Broodstock Program (MBP) through Oregon State University has been selecting *C. gigas* families for yield and survival in estuaries along the West Coast since 1996, with seven generations of oyster families produced so far [46]. More recently, MBP has begun identifying families for resistance to OsHV-1 and its variants based on survival when exposed to OsHV-1 in the field and OsHV-1 µvar in the laboratory [47]. Of these, ten high- and ten low-surviving families were chosen for use in the present study. The goal of this study was to investigate the resistance of *C. gigas* juveniles to three variants of OsHV-1: a California reference OsHV-1 (OsHV-1 TB), an Australian µvar (AUS µvar), and a French µvar (FRA µvar). The AUS µvar and FRA µvar isolates used in this study are the same as those used in previous comparative studies [44,45].

## 2. Results

### 2.1. Inoculum

Groups of sub-adult oysters were injected with one of the three OsHV-1 variants and incubated to allow viral shedding for 24 h, which resulted in varied concentrations of OsHV-1 for each inoculum. For all three treatments, each petri dish was inoculated with 60 mL of corresponding exposed seawater (from the sub-adult oysters), resulting in exposure to 2.51 × 10^8^, 1.09 × 10^7^, and 7.67 × 10^6^ total copies of OsHV-1 DNA for the FRA µvar, the AUS µvar, and OsHV-1 TB, respectively. Consequently, oysters exposed to the French µvar were exposed to 23 and 33 times more OsHV-1 viral DNA copies than the AUS µvar and OsHV-1 TB, respectively, on day zero.

### 2.2. Mortality

**Figure 1:**
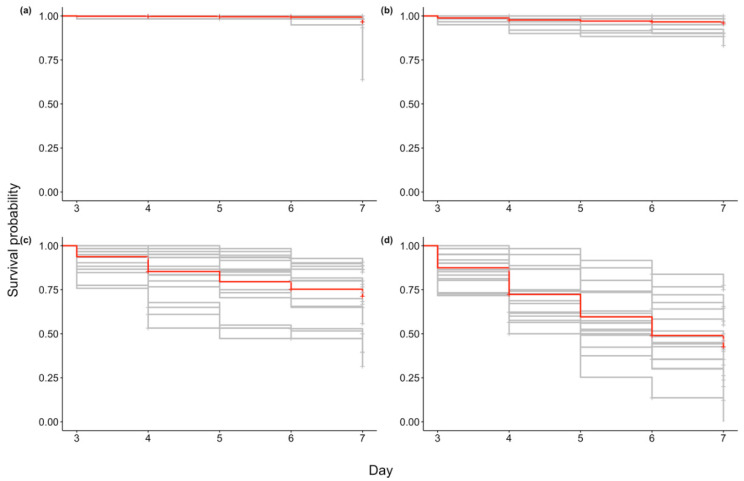
Kaplan–Meier survival curves for all 19 oyster families (excluding family 60) exposed to (**a**) filtered seawater (controls), (**b**) OsHV-1 TB, (**c**) AUS µvar, and (**d**) FRA µvar (*n* = 60 oysters/family). Red lines indicate average survival across all families for each treatment.

**Figure 2:**
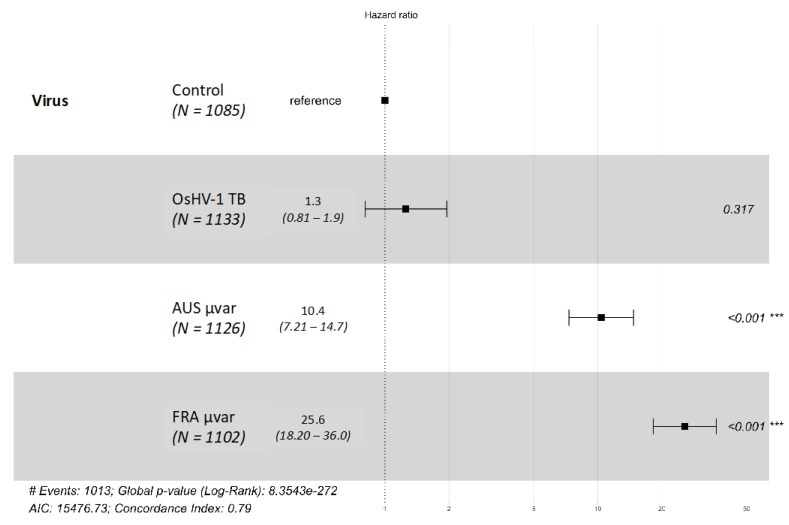
Cox proportional hazards plot for Kaplan–Meier survival analysis for 19 Pacific oyster families exposed to OsHV-1 TB, AUS µvar, and FRA µvar as compared to the controls. *** *p* < 0.001.

**Figure 3:**
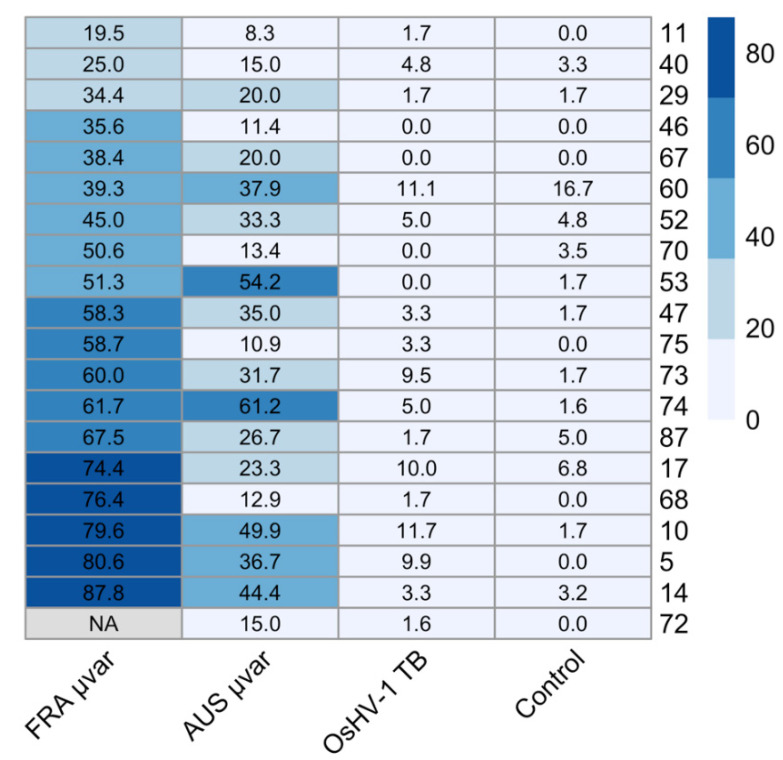
A heat map representing mean mortality for all 20 oyster families (right) exposed to either the FRA µvar, AUS µvar, OsHV-1 TB or controls (filtered seawater).

Survival analysis based on daily mortality counts from day three to day seven indicated that exposure to either the FRA µvar or AUS µvar caused lower survival probability and increased risk of mortality than exposure to OsHV-1 TB and the controls, and exposure to the FRA µvar caused the highest mortality of all three variants (Figure 1; Log-Rank χ^2^ = 1295, df = 3, *p* < 0.0001). Family 60 had higher mortality in the controls than when exposed to OsHV-1 TB (7.4% higher), and therefore was removed from further analysis (see Table S1). Kaplan–Meier survival curves were generated for each virus pooled across all remaining 19 oyster families. The survival probability of oysters when exposed to OsHV-1 TB (96.0 ± 0.6%) was not significantly different than the controls (96.6 ± 0.6%; *p* > 0.05). The survival probability for oysters exposed to the FRA µvar (42.6 ± 1.5%) or the AUS µvar (71.3 ± 1.4%) was lower than those exposed to OsHV-1 TB and the controls (*p* < 0.0001). Oysters exposed to the FRA µvar had lower survival probability than those exposed to the AUS µvar (*p* < 0.01). Hazard ratios indicated that exposure to the FRA µvar or AUS µvar increased risk of mortality compared to the controls, while exposure to OsHV-1 TB did not increase risk of mortality (Figure 2). Among all 19 oyster families, four were identified as having an increased survival probability compared to all other families when exposed to the AUS µvar or FRA µvar (survival probability 65–91%; *p* < 0.05) and similar to each other (*p* > 0.05; Figure 3); families 11, 40, 46, and 72 (although family 72 was only exposed to OsHV-1 TB and the AUS µvar).

**Figure 4:**
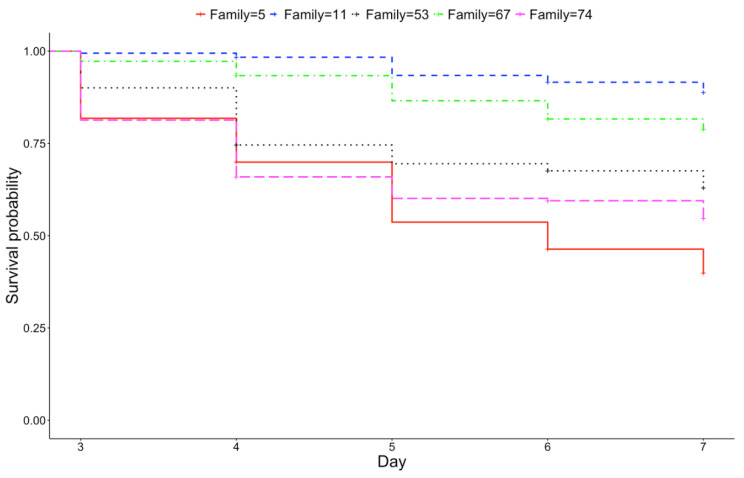
Kaplan–Meier survival curve for the five families initially predicted to have high or low survival (excluding family 60) averaged across exposure to OsHV-1 TB, AUS µvar and FRA µvar (*n* = 180 oysters/family on day 1).

Subsets of high-performing (11, 53, 67) and low-performing (5, 74) families based on a previous study [47] were chosen for more in-depth analysis, as well as qPCR analysis (see 2.3). When averaged across each virus, these families had a range of survival (Figure 4; Log-Rank χ^2^ = 111, df = 4, *p* < 0.0001). The survival probability of family 11 (88.8 ± 2.5%) was greater than all other families (*p* < 0.05), followed by family 67 (78.8 ± 3.2%; *p* < 0.0001). The survival probability of family 53 (63.0 ± 3.8%) was greater than family 5 (39.0 ± 4.5%; *p* < 0.001), and the survival probability of family 74 (54.6 ± 3.9%) did not significantly differ from family 53 or family 5 (*p* > 0.05).

**Figure 5:**
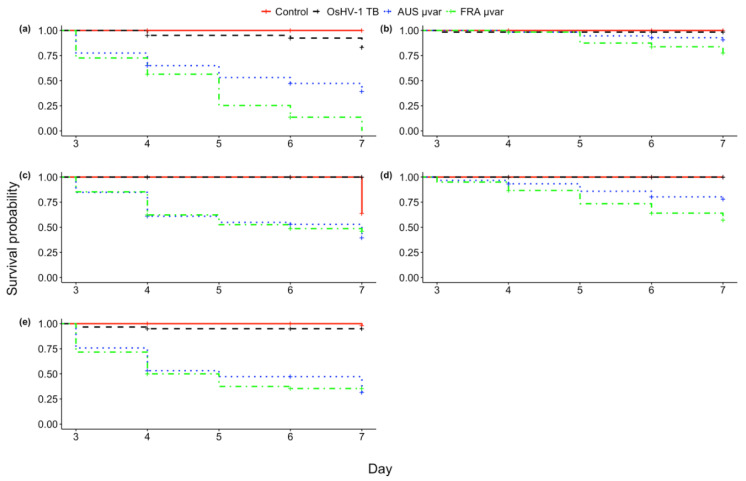
Kaplan–Meier survival curves for (**a**) family 5, (**b**) family 11, (**c**) family 53, (**d**) family 67 and (**e**) family 74 when exposed to filtered seawater (control), OsHV-1 TB, AUS µvar or FRA µvar (*n* = 60 oysters/treatment on day one).

Individual survival probability of each of these five families varied depending on the virus to which they were exposed (Figure 5; Log-Rank for family 5, 11, 53, 67, 74, χ^2^ = 77.6, 21.4, 63.3, 52.7, 103, respectively, df = 3, *p* < 0.0001). For each individual family, survival probability when exposed to OsHV-1 TB did not significantly differ from the controls (*p* > 0.05), except for family 53, which had a higher survival probability when exposed to OsHV-1 TB than the controls (*p* < 0.001). However, each oyster family experienced lower survival probability when exposed to either the FRA or AUS µvar (9.3–100% lower) relative to the controls and OsHV-1 TB (*p* < 0.01), except Family 11, where survival probability when exposed to the AUS µvar or OsHV-1 TB was equivalent (*p* > 0.05). For families 5 and 67, exposure to the FRA µvar caused 40% and 21% lower survival probabilities than exposure to the AUS µvar, respectively (*p* < 0.05). Family 11, 74, and 53 all had equal survival probability when exposed to either µvar (*p* > 0.05).

Survival of all 20 oyster families was positively correlated among all viruses. The survival of oysters correlated between those exposed to OsHV-1 TB and both the AUS µvar (Spearman rank correlation; rho = 0.47, *p* = 0.03) and the FRA µvar (rho = 0.47, *p* = 0.04). Oyster survival also correlated between the AUS µvar and the FRA µvar (rho = 0.50, *p* = 0.03). The survival of oysters exposed to OsHV-1 TB in the present study also correlated with survival to OsHV-1 TB in the field (rho = 0.50, *p* = 0.04) and survival to the same FRA µvar in another laboratory study (rho = 0.56, *p* = 0.01) by Divilov et al. [47]. There was no significant correlation between the survival of oysters exposed to the FRA µvar in the present study and survival to OsHV-1 TB in the field or the FRA µvar in the laboratory study (rho = 0.141 and rho = 0.45, respectively; *p* > 0.05) by Divilov et al. [47]. 

### 2.3. qPCR of Dead and Live Oysters

Subsets of high-performing (11, 53, 67) and low-performing (5, 74) families based on a previous study [47] were chosen for qPCR analysis. OsHV-1 was detected in all dead oysters that were assayed on days four and six using qPCR as a proxy for infection. Average OsHV-1 viral copy number for select dead oysters exposed to OsHV-1 TB (*n* = 5), the AUS µvar (*n* = 20), and the FRA µvar (*n* = 22) were (mean ± SE) 3.83 × 10^7^ ± 3.04 × 10^7^, 2.11 × 10^7^ ± 3.51 × 10^6^, and 1.22 × 10^7^ ± 2.32 × 10^6^ copies per oyster, respectively. These loads are equivalent to a 5.00 and 1.94 times increase in OsHV-1 loads per dead oyster relative to original inoculum added for OsHV-1 TB and the AUS µvar, respectively, and a 20.57 times decrease from the original FRA µvar inoculum.

**Figure 6:**
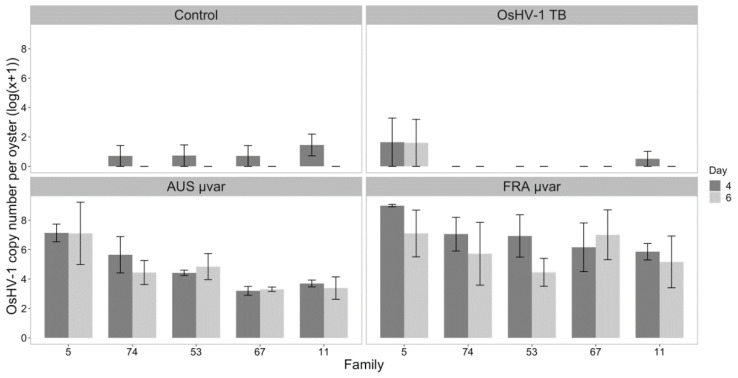
OsHV-1 copy number (log_10_(*x* + 1)) in live oysters (*n* = 6 oysters/day) for each oyster family exposed to filtered seawater (Control), OsHV-1 TB, AUS µvar, and FRA µvar on days four and six. Oyster families are in order from lowest-highest survival probability as an average across all viruses. Error bars represent standard error.

Of these five families, live oysters exposed to either the FRA or AUS µvar accumulated higher OsHV-1 viral copy numbers (Figure 6, Wilcoxon Rank Sum Test *p* < 0.0001; all comparisons) compared to those exposed to OsHV-1 TB and controls, which were similar (Overall Kruskal–Wallis Rank Sum Test χ^2^ = 90.79, df = 3; *p* < 0.0001; Wilcoxon Rank Sum Test for TB and controls, *p* = 0.068). Higher copy numbers were detected in oysters exposed to the FRA µvar compared to the AUS µvar (Wilcoxon Test, *p* = 0.0047). OsHV-1 infection in live oysters did not differ among families (Kruskal–Wallis, χ^2^ = 6.673, df = 4, *p* > 0.05) or between days (Kruskal–Wallis χ^2^ = 1.14, df = 1, *p* > 0.05). 

### 2.4. Water Samples

**Figure 7:**
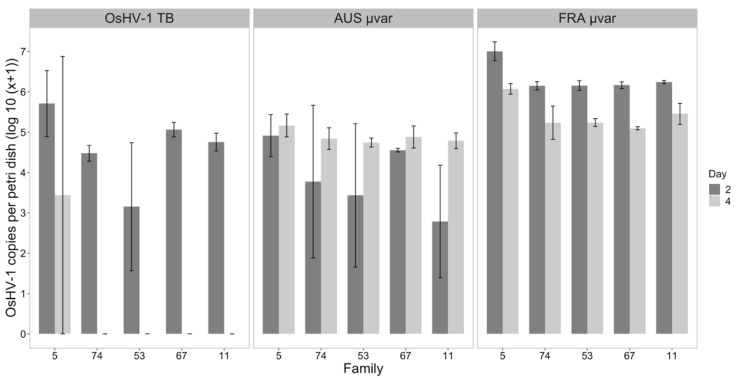
OsHV-1 copy number (log_10_ (*x* + 1)) per petri dish (60 mL total/petri dish) of the exposed water of each oyster family exposed to OsHV-1 TB, AUS µvar, and FRA µvar on days two and four (*n* = 3). Oyster families are in order from lowest-highest survival probability as averaged across all viruses. Error bars represent standard error.

The same subset of five families was used to investigate the concentration of OsHV-1 DNA in petri dish water. The presence of OsHV-1 in water of oysters exposed to the FRA and AUS µvar on days four and six indicated continued viral shedding, as opposed to water of oysters exposed to OsHV-1 TB, which greatly decreased in OsHV-1 DNA concentration on day 6. The best-fit linear model included a fixed effect for family, virus, day, and the interaction for virus by day (Figure 7; see Table S2). On day two, higher OsHV-1 copy numbers were detected in water for the FRA µvar as compared to the AUS µvar (*p* < 0.0001) and OsHV-1 TB (*p* < 0.0001); the AUS µvar and OsHV-1 TB were similar on Day 2 (*p* < 0.05). On day four, the OsHV-1 DNA quantified in the water of oysters exposed to FRA and AUS µvar did not differ (*p* < 0.05), but water for families exposed to OsHV-1 TB had 52,500 and 11,100 times fewer viral copies than FRA and AUS µvar (*p* < 0.0001). When averaged across all viruses, increased copy numbers were detected in the water of Family 5 as compared to Family 11 (0.05), Family 53 (*p* < 0.01), Family 74 (*p* < 0.05); no other significant differences were detected between families.

## 3. Discussion

This is the first study to directly compare the survival and infection of Pacific oysters to OsHV-1 µvars with a non-µvar, OsHV-1 TB, using experimental infections in the laboratory. Exposure of oysters to either the FRA µvar or the AUS µvar caused significantly lower survival probabilities, higher cumulative mortality, and increased the risk of death compared to exposure to OsHV-1 TB. The survival probability of oysters exposed to OsHV-1 TB did not differ from the controls. Low levels of OsHV-1 were detected in some control plates. A routine health exam using a random sample of the sourced juvenile oysters found no detectable OsHV-1 DNA via PCR (see Section 4.1.1), and therefore these positive controls were likely due to aerosolization of viral particles during inoculation of the OsHV-1 variants or during experimental handling. Despite viral DNA presence in control samples, exposure to the FRA or AUS µvar caused significantly higher mortality than observed in the controls. Furthermore, all control oysters sampled on day six were negative for OsHV-1, indicating that the minimal exposure of control plates to the virus or its DNA did not result in replication or spread among oysters. 

Prior to the emergence of OsHV-1 µvar, laboratory transmission of OsHV-1 to juvenile oysters was never achieved. Transmission of OsHV-1 between larval oysters was well established [48,49], and transmission from juveniles to larvae using OsHV-1 TB was accomplished [50]. In the present study, infection of oysters with OsHV-1 TB was not successful for every family, as indicated by the lack of mortality and viral DNA within live oyster tissue and water. We recently used the same OsHV-1 TB inoculum from the present study to infect via injection (>10^7^ copies/µl of OsHV-1 in dead animals) and cause mortality (70% mortality) of susceptible sub-adult oysters over four days [51]; no other infections were identified with histology. In the current study, all dead juvenile oysters exposed to OsHV-1 TB contained high concentrations of virus (mean ± se; 3.83 × 10^7^ ± 3.04 × 10^7^). Taken together, this suggests that the viral inoculum used was virulent, but did not cause infection in all oysters. It is possible that the initial viral dosage was not high enough to cause infection. Alternatively, the families tested may be less susceptible to OsHV-1 TB, supported by the observed concordance in survival between oysters in the present study and those exposed to OsHV-1 TB in the field (rho = 0.50) by Divilov et al. [47]. Although these oyster families may have decreased susceptibility to OsHV-1 TB, they still succumbed to mortality and accumulated high virus concentrations when exposed to either the FRA or AUS µvar.

Survival to a pathogen can occur through three mechanisms: inability to become infected (decreased susceptibility), the ability to control a pathogen once infected (increased resistance), or a lack of disease despite infection (increased tolerance) [52,53]. The high OsHV-1 copy numbers detected in potentially surviving oysters in this study points to tolerance in lieu of resistance. As such, the accumulation of viruses in asymptomatic animals could be devastating to nearby susceptible oyster families. OsHV-1 has been known to accumulate and persist within the tissue of asymptomatic adult oysters [33,54,55], and it is hypothesized that OsHV-1 in these individuals can be passed via vertical transmission to offspring [54,56,57]. Asymptomatic oysters that survive an OsHV-1 infection may also act as reservoirs by harboring latent infections that are reactivated at a later time, likely when exposed to stressful conditions such as an increase in temperature [44,56]. OsHV-1 in larval oysters can also be transmitted between species [49], and therefore tolerant animals pose a threat to other species nearby that are susceptible to OsHV-1 with few mechanisms for tolerance or resistance. Accumulation and persistence of virus in asymptomatic oysters also increases the risk of virus spread through the transport of animals if proper screening does not occur. Therefore, it would be ideal that a resistant oyster family should accomplish survival through decreased susceptibility or increased resistance, identified as lack of viral replication within the host.

Oyster families in the present study that appeared to be “resistant” based on potential high survival to the FRA or AUS µvar still accumulated high amounts of OsHV-1 DNA in live tissue. Due to the small size of oysters in this study, we are able to present our data in OsHV-1 copies per oyster with additional data on copies/ng of DNA (see Table S3). Based on a subset of oysters that were weighed (frozen tissue weights), we approximate a wet tissue weight of ~5–10 mg of tissue per oyster. The mean concentration of OsHV-1 in surviving oysters exposed to any variant was 3.53 × 10^8^ ± 1.63 × 10^8^ copies per oyster (mean ± se), or ~35–70 million copies per 5–10 mg wet weight, which is similar to susceptible lines in other studies [40,41,44,47]. This concentration was also higher than OsHV-1 concentrations detected in dead oysters exposed to the FRA and AUS µvar in the present study (mean ± se; 1.64 × 10^7^ ± 2.13 × 10^6^ copies per oyster; ~1.5–3 million copies per 5–10 mg we weight). The threshold of increased risk of death from OsHV-1 µvar infection is considered 8.8 × 10^3^ copies/mg of oyster tissue [55], and when concentrations are >10^5^ copies/mg of tissue, the OsHV-1 µvar is considered the principal cause of death [40]. Concentrations of OsHV-1 DNA in both live and dead tissue of potentially high surviving oyster families was well above the 10^5^ copies per mg threshold. Thus, it is possible that a longer experimental time in this study would have resulted in increased mortality in these surviving individuals with high copy numbers [44,45]. These results highlight the benefit of using qPCR as a proxy for viral infection to ensure true resistance when testing oyster families for resistance to OsHV-1 variants.

Many oyster families varied in survival depending on which variant they were exposed to, and high survival in relation to one variant did not always result in high survival in relation to the other two. These results demonstrate the importance of testing oyster families to multiple variants of OsHV-1 prior to determining resistance. Oyster families 11, 40, 46, and 72 did show high survival (>65%) when exposed to all three variants (although family 72 was only exposed to OsHV-1 TB and the AUS µvar). In previous studies, oyster lines resilient to a French OsHV-1 reference variant have been shown to also display resistance to OsHV-1 µvars [39,41]. These oysters were able to stop infection progression and remove an OsHV-1 µvar from their tissues [39]. In contrast, weak or insignificant correlations were found between breeding values for survival of oysters exposed to OsHV-1 TB in the field and the same FRA µvar used in the present study in a laboratory study [47], although this lack of correlation could be due to a variety of factors, including potential differences in experimental conditions in the field and differences in methods between the laboratory and field study [45]. In the present study oyster survival did correlate between oysters exposed to OsHV-1 TB and either the AUS or FRA µvar (rho = 0.47 for both) and between the survival of oysters exposed to the FRA µvar and to the AUS µvar (r = 0.50). Further identification and testing of oyster families with strong survival correlations among variants could produce lines resistant to both OsHV-1 µvars and non-µvars. The recent emergence and potential spread of an OsHV-1 µvar in southern California [30] calls for a proactive approach to management, and testing for resistance to multiple variants, especially µvars, will continue to be critical when developing OsHV-1-resistant lines. 

Our results also highlight key questions regarding the pathogenesis of different OsHV-1 variants that will help improve future research and better inform breeding programs. Variations in viral shedding rate likely led to different initial viral doses for each treatment (2.51 × 10^8^, 1.09 × 10^7^, and 7.67 × 10^6^ total copies of the FRA µvar, the AUS µvar, and OsHV-1 TB, respectively). Each virus was allowed to incubate in donor oysters for as long as possible; however, due to time constraints, the AUS µvar and OsHV-1 TB did not incubate long enough to shed the same amount of virus as the FRA µvar. The inoculum for the FRA µvar contained the highest viral dose, suggesting that donor juvenile oysters shed this variant at a faster rate than the AUS µvar, which in turn was shed faster than OsHV-1 TB. Additionally, the concentration of OsHV-1 in petri dish water on day four of the experiment was similar for oysters exposed to the FRA and AUS µvar, despite differences in initial viral doses and greater viral loads in the tissue of live juvenile oysters exposed to the FRA µvar. By day four, less oysters were in the petri dishes exposed to the OsHV-1 FRA µvar (due to mortality) indicating that fewer oysters contributed similar copy numbers to the water. Viral shedding is an important mechanism for viral spread and key in understanding the pathogenesis of each variant: in other aquatic herpesviruses such as channel catfish virus (CCV), koi herpesvirus (KHV), and salmonid herpesvirus-3 (EEDV), viral shedding is key to dispersal [58,59,60]. To better understand OsHV-1 viral shedding rates, experiments may need to quantify the rate of individual oysters exposed to each microvariant. 

Our study also highlights the need to consider differences in virulence among variants when testing oyster families for resistance. Oysters exposed to the FRA µvar experienced the highest mortality compared to the other two variants; however, dead oysters for all three variants contained the same order of magnitude of OsHV-1 DNA. Burge et al. [44] found comparable results, where oysters exposed to the same FRA µvar experienced statistically higher mortality (97.5% vs. 90% mortality) than those exposed to the same AUS µvar, though virus concentrations within both live and dead oyster tissues were similar. Our results also suggest a maximum amount of virus per oyster as total OsHV-1 DNA copies per dead oyster were similar between all three variants despite being exposed to different doses. Combined, these results support our notion that further research is necessary to characterize differences in virulence among variants, specifically the µvars. Very little is known about the pathogenesis of OsHV-1 or OsHV-1 µvars; studies regarding shedding rates, the minimum number of virions needed to induce mortality and variations of these characteristics among different oyster stocks and viral variants will be crucial to understanding OsHV-1 as new variants continue to emerge. 

This study was conducted under constant conditions, although virus virulence and resulting oyster survival could be affected by environmental conditions. Summer mortality of oysters can result from a multitude of factors, including infection with OsHV-1 and *Vibrio* spp., changes in temperature and salinity, and age of the oysters [61]. Specifically, increases in temperature have been reported to correlate with OsHV-1 outbreaks and subsequent mortalities. Increases in temperature are considered the most important factors influencing mortality by oyster farmers in Australia [62]; however, the temperature limits for OsHV-1 outbreaks are variable throughout its geographic range. Outbreaks occur from 18 to 25 °C in Australia, and 16 to 24 °C in France and Tomales Bay, CA, and often correlate with increased means or maximums for the year in seawater temperature [16,17,26,33,40,63,64]. Extremely high temperature (29 °C) may even mitigate infection [65]. The survival of oyster families could, therefore, vary in different temperatures and with exposure to different variants. 

Oyster size at time of infection may also play a role in survival and is important to consider when selecting lines for resistance. Larger oysters are known to be less susceptible than smaller oysters [16,33,40]; therefore, an oyster family resistant to OsHV-1 in the juvenile form may be more susceptible at larval stages. It has been previously demonstrated that oysters selected for resistance to a French OsHV-1 variant as juveniles and adults also show decreased mortality compared to unselected oysters as larvae; however, this study was conducted at a constant temperature for only one oyster family [66]. Larvae in this family still experienced mortality when exposed to OsHV-1 and accumulated OsHV-1 DNA in their tissues, which may become latent and spread at a later life stage [66]. Furthermore, whether exposed larvae that survive can settle and continue to develop remains unknown [66]. It will be important to select for families that demonstrate resistance (versus tolerance) to OsHV-1 across multiple life stages and are resilient to environmental stressors, to ensure survival when exposed to OsHV-1 variants in the field [47].

## 4. Materials and Methods

### 4.1. Oysters Challenged with OsHV-1

#### 4.1.1. Oysters

Juvenile oysters (*n* = 20 families, 6–8 mm, see Table S4) were shipped overnight (25 June 2018) on ice from the Molluscan Broodstock Program (MBP) at Oregon State University to the University of Arizona Aquaculture Pathology Laboratory (UA APL). Prior to being shipped to France [47], a routine health exam of 175 juvenile oysters randomly chosen from these families was conducted by AquaTechnics Inc. [67]; no OsHV-1 was detected via PCR, nor were there any other health anomalies. The chosen families for the present study were MBP “Cohort 27” (spawned on 28 February 2018) and were chosen based on previous exposures to an isolate of OsHV-1 µvar in France [47]; ten were high-surviving (~75–85% survival) and ten were low-surviving (~0–6% survival) families. Once received, oysters were kept in aerated 0.22 µm filtered seawater (FSW) from Newport, Oregon (from the MBP hatchery) and gradually brought to 22 °C. Juvenile oysters were held for a minimum of 48 h prior to experiments. Oysters were fed ad libitum Caribbean *Isochrysis* (C-ISO) until 24 h before trials began.

Sub-adult oysters to be used as donor oysters for OsHV-1 inoculum (~20 mm) were shipped overnight (25 June 2018) on ice from Hog Island Oyster Company, Humboldt Bay, California: an area known to be free of OsHV-1 [68]. The oyster stock used is known to have a low survival in the presence of OsHV-1 TB in Tomales Bay, California [69]. Oysters were placed in a tank containing 4 L of 30 ppt artificial seawater (ASW) made with Crystal Sea(R) Marine Mix 150 gallon (~567 L) mixture (Marine Enterprises International) dissolved in distilled water. All oysters were held in water containing antibiotics (15 mg/mL streptomycin and 150 units/mL penicillin) [50].

#### 4.1.2. Inoculum

Experiments were conducted with permission from the Arizona state veterinarian and the United States Department of Agriculture Animal and Plant Health Inspection Service (USDA-APHIS). The inocula were created following methods of Kirkland et al. [70] and held at −80 °C at the UA APL (same inocula as [44,45]). Isolates were obtained from an AUS µvar collected at George’s River, Australia [44,45,70], a FRA µvar from Marennes-Oléron Bay [44,45], and OsHV-1 TB from Tomales Bay, California. Inoculum from Tomales Bay, California was created from oysters collected after a mortality event in 2017 and held at −80 °C following methods as described in Burge and Friedman [50] and Kirkland et al. [70] to produce 0.22 µm filtered homogenates, and frozen using cryopreservation methods [44,70].

**Figure 8:**
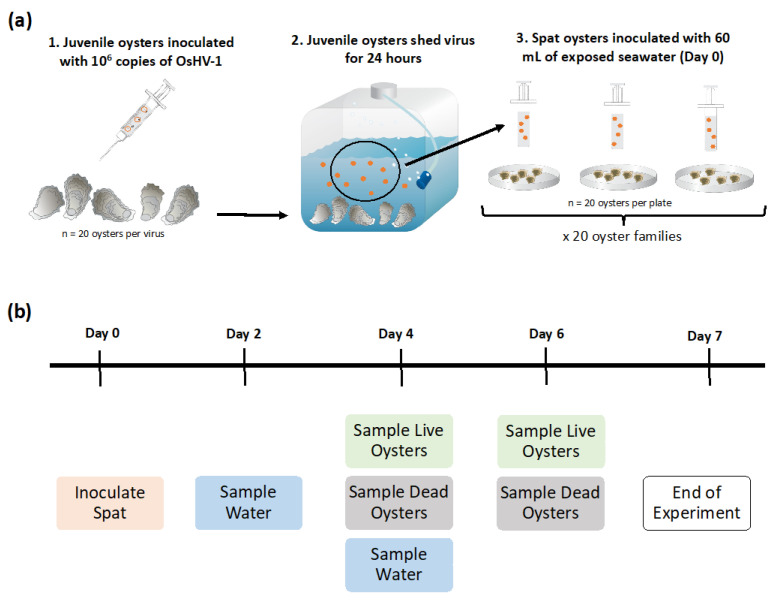
(**a**) Experimental methods and (**b**) timeline of sampling. Methods were repeated identically for all treatments (Ostreid herpesvirus-1 (OsHV-1) Tomales Bay (TB), French (FRA) µvar and Australian (AUS) µvar), and controls were sham-inoculated with filtered seawater (images by Saxby, Tracey and Wicks, Caroline, Integration and Application Network, University of Maryland Center for Environmental Science, ian.umces.edu/imagelibrary/).

These inocula were then used to infect 20 sub-adult diploid *C. gigas* via injection into the adductor muscle of 1 × 10^6^ copies of either FRA µvar, AUS µvar, or OsHV-1 TB (Figure 8). Injection was staggered in 24 h intervals to avoid contamination, beginning with the FRA µvar, followed by the AUS µvar, then OsHV-1 TB. Oysters were placed back into their original aquarium with a 50/50 mixture of 0.22 µm FSW from Newport, Oregon and ASW. Injected oysters were left in their corresponding tanks for approximately 24 h to allow for viral replication and shedding. Total viral concentration of the original homogenate and water that contained shed virus (exposed water) was determined by extracting 200 µL of corresponding sample followed by an OsHV-1 specific qPCR assay (see Section 4.3). 

#### 4.1.3. Infection of Juvenile Oysters

Three replicates of juvenile oysters (*n* = 20) were placed into petri dishes for each of 20 *C. gigas* families (see Table S1), except for family 5 which only had two replicates for OsHV-1 TB and the AUS µvar, and family 72, which was not exposed to the FRA µvar. This set-up was repeated for each treatment (FRA µvar, AUS µvar, and OsHV-1 TB), resulting in 58 petri dishes for OsHV-1 TB and the AUS µvar and 57 for the FRA µvar. On day zero, each petri dish was inoculated with 60 mL of exposed water (see Section 4.1). On day zero for each treatment, one additional petri dish containing 20 juvenile oysters for each family was sham-inoculated with ASW as a control. This resulted in three control replicates for each family, except family 5, where only one control was used. All replicates were kept at 22 °C for a total of seven days.

### 4.2. Oyster and Water Sampling

#### 4.2.1. Mortality Counts

Starting on day three, petri dishes were checked daily for mortalities as indicated by a gaping oysters that did not close when lightly probed. The numbers of live and dead oysters were counted, and dead oysters were removed.

#### 4.2.2. Sampling of Water and Oysters

The MBP program had previously determined ten high-surviving and ten low-surviving families when exposed to OsHV-1 µvar FRA [47]. Three of these high-surviving and three low-surviving families were chosen for more in-depth analysis (see Table S1). On days four and six, two live and up to three dead oysters from each replicate were collected for these six families. Water samples (200 µL) were also collected from each of these six families on days two and four. All samples were initially frozen at −20 °C to inactivate the virus [71] then stored at −80 °C until further analysis.

### 4.3. Sample Extraction and OsHV-1 Detection via qPCR

To obtain OsHV-1 viral copy number in resulting samples, 200 µL of the viral inoculum and exposed water, as well as water samples taken during the exposure experiment, were extracted using the Zymo Quick DNA Miniprep Plus Kit following the biological fluids protocol. Both live and dead sampled oysters were individually dissected from their shells and the whole body (~5–10 mg) was extracted using the Zymo Quick DNA Miniprep Plus Kit following the solid tissue protocol. 

An OsHV-1-specific qPCR test was run on all extracted homogenates, inocula, oyster tissues, and water samples to quantify total OsHV-1 viral gene copy numbers as a proxy for viral infection. The qPCR protocol followed methods outlined in Burge and Friedman [50], targeting the OsHV-1 ORF 100/catalytic subunit of a DNA polymerase using the primers ORF 100F (5′-TGA TGG ATT GTT GGA CGA GA-3′) and ORF 100R (5′-ATC ACA TCC CTG GAC GCT AC-3′). All qPCRs were performed on an Applied Biosystems^®^ 7500 Fast Real-Time PCR System. The 20 µL reaction consisted of 10 µL of SYBR Green Master Mix, 15 µg of BSA, 5.9 µL of PCR water, 400 nM of forward and reverse primer, and 2 µL of sample DNA. The thermocycler protocol consisted of an initial denaturation step at 95 °C for 20 s, followed by 40 cycles of 95 °C for 3 s and 60 °C for 30 s, and a final melt curve analysis consisting of continuous fluorescence monitoring during a 20 min temperature ramp to 95 °C that was held for 15 s. All qPCRs included a standard curve generated from dilution of a plasmid constructed in our laboratory from 3 × 10^7^ down to 3 copies per reaction, the limit of detection for this assay [50]. A melting temperature peak shift ± 1 °C from the standard controls was set as a cut off for species-specific amplification. All standards and samples were run in duplicate. The concentration of DNA in ng/µL was determined for all positive samples using a Quant-IT ^™^ PicoGreen dsDNA Assay Kit (Molecular Probes) with fluorescent intensity (485 nm excitation, 535 nm emission) measured using a Molecular Devices FilterMax F5 microplate reader with SoftMax Pro 64 software (Molecular Devices, San Jose, CA, USA). 

Oyster tissue samples that were negative for OsHV-1 DNA were run in a second qPCR to ensure the presence of amplifiable DNA by amplifying the 18S gene of *C. gigas* using the primers CG 18S F (5′-CAG CGA AAG CAT TTG CCA AG-3′) and CG 18S R (5′CAC CCA CCG AAT CAA GAA AGA G 3-’) [50]. The master mix concentrations are identical to those mentioned above, with a thermocycler protocol as follows: 95 °C for 20 s followed by 35 cycles of 95 °C for 3 s and 55 °C for 30 s, and a final melt curve analysis. All samples run were positive for 18S DNA.

### 4.4. Statistical Analysis

All statistical analyses were performed using R version 4.0.2 (The R Foundation, Vienna, Austria) [72]. An alpha of 0.05 was set for all significance tests. All figures were generated using the package ggplot2 [73], except Figure 3, which was generated using the package pheatmap [74]. A survival analysis was conducted using the survival [75] and survminer [76] packages to generate Kaplan-Meier curves with log-rank chi-square tests and Cox proportional hazard ratios to investigate differences in survival probability of oysters exposed to each virus treatment. We also investigated the survival probability of the five (family 60 was excluded, see 2.2) high- or low-surviving families (averaged across all viruses), and the survival probability of the individual five high- or low-surviving families among viruses and controls.

To investigate the effects of treatment on concentrations of OsHV-1 in tissues and water, we used linear models. All combinations of linear models were run to determine which factors contributed to accumulation of qPCR copy number in live oysters or water, with fixed effects for virus, oyster family, day, and their interaction. All qPCR data were log_10_ (*x* + 1) transformed to normalize them prior to analysis. The package MuMIn [77] was used to fit all combinations of models from a global model. The best fit model was chosen based on minimizing the Akaike Information Criterion with a correction for the small sample size (AICc). The diagnostics of model fit were examined for each model and satisfied, except for the linear model to test the effects of treatment on concentrations of OsHV-1 in tissues. Differences among treatments and families for tissue concentrations were thus tested individually with a Kruskal-Wallis test (*p* < 0.05) followed by a multiple comparison using the Wilcoxon Rank Sum Test with a “holm” probability adjustment [78].

Correlations were run between survival data in the present study and the corresponding oyster families exposed to the FRA µvar via bath exposure and OsHV-1 TB in the field by Divilov et al. [47]. Correlations were run with the cor.test function using the Spearman method due to non-normality in the data. All data and statistical code are available on figshare [79].

## 5. Conclusions

We examined the resistance of 20 oyster families to three variants of OsHV-1: a French µvar, an Australian µvar, and a non-µvar from Tomales Bay, California. Importantly, we have defined the desirable breeding trait for disease resistance as increased survival and decreased susceptibility or a lack of viral replication within the host. Our findings highlight the importance of the inclusion of qPCR viral loads as part of breeding programs focused on OsHV-1 resistance to identify tolerant oyster families, as increased survival does not always indicate resistance. OsHV-1 has historically spread through unclear sources. Minimizing the spread within and between species and geographic regions is key to mitigating the impact of this disease, and asymptomatic reservoir populations of oysters threaten the transmission of this virus. Our data demonstrate the importance of testing oyster families against multiple OsHV-1 variants, as high survival in relation to one variant does not always determine high survival in relation to another. Although no family in the present study showed resistance to all three variants, further breeding of oyster families with correlations in survival among multiple variants is promising for the development of multi-variant resistant oysters. Finally, we pose multiple areas of improvement for OsHV-1 research. These include investigations into variant shedding rates and the minimum number of virions needed to induce infection, as well as testing the effects of multiple variants under different environmental conditions and oyster life stages to ensure that laboratory results translates to resistance in the field. As new variants of OsHV-1 continue to emerge and devastate Pacific oyster populations, persistent research in these areas will be crucial to maintaining this economically and ecologically important species.

## Supplementary Materials

The following are available online at https://www.mdpi.com/2076-0817/9/12/1057/s1, Table S1: Mean mortality for all 20 oyster families, Table S2: Linear model results for water qPCR, Table S3: Copies of OsHV-1 DNA per ng of oyster DNA, Table S4: Average oyster sizes.
